# A comprehensive review on the protein and lipid quality of marine scallops

**DOI:** 10.3389/fnut.2026.1872875

**Published:** 2026-07-29

**Authors:** Junlong Xu, Zhou Wu, Xin Li, Karsoon Tan

**Affiliations:** 1 College of Marine Science, Guangxi Key Laboratory of Marine Environment Disaster Processes and Ecological Protection Technology, Beibu Gulf Ocean Development Research Center, Pinglu Canal and Beibu Gulf Coastal Ecosystem Observation and Research Station of Guangxi, Beibu Gulf Marine Ecological Environment Field Observation and Research Station of Guangxi, Beibu Gulf University, Qinzhou, Guangxi, China; 2 Fangchenggang Vocational and Technical College, Fangchenggang, Guangxi, China

**Keywords:** amino acids, fatty acids, global, marine scallops, proximate composition

## Abstract

The advent of positive socioeconomic changes and rising incomes has precipitated a dramatic increase in demand for luxury seafood, including scallops. The nutritional quality of scallops has been reported for different species from various geographical regions, but this information is not well organized. In this context, the objective of this study was to conduct a systematic evaluation of the proximate composition, amino acid quality, and fatty acid quality of scallops on a global scale. The protein and lipid composition of scallops exhibited significant variability among studies. Scallops are a source of high-quality protein and lipids, with fatty acid profiles that are notably superior. While certain scallops are known to contain all essential amino acids in sufficient amounts, valine frequently emerges as the primary limiting essential amino acid in the majority of scallops. With respect to the composition of fatty acids, scallops exhibit a notable abundance of n-3 long-chain polyunsaturated fatty acids (LC-PUFA). The content of EPA + DHA in most scallops exceeds 20% of total fatty acids, and in some cases reaches as high as 50%. The lipid quality indices (n-3/n-6 and PUFA/SFA ratios) of scallops are generally higher than recommended values, with the exception of a few documented cases with unusually low PUFA/SFA ratios. The findings of this study offer consumers detailed nutritional information on scallops and serve as important guidance for developing aquaculture and selective breeding programs for scallops.

## 1 Introduction

In recent decades, socio-economic advancements, particularly increasing income levels and rapid urbanization, along with growing awareness of the health benefits of dietary protein, have significantly increased the global demand for animal protein, which is projected to reach 500 million tonnes annually by 2050 (1). However, the increasing demand is not uniform for all animal protein sources, with a higher increase in demand for luxury proteins (such as seafood) than for staple protein sources (such as pork and chicken) (2, 3). This is due to the growing number of reports associating luxury foods with high and balanced nutrition that promotes health and healthy aging (4, 5). Consequently, this growing preference for luxury animal proteins places additional pressure on production systems.

Among luxury proteins, scallops are a popular high-end seafood in Asia, Europe, and the United States of America (6, 7), where they are reserved for hotels and high-class restaurants. To date, scallops are the third most produced bivalve from aquaculture, after oysters and mussels, with scallop production having shown an increasing trend over recent decades (8). In terms of economic value, the global scallop market in 2020 was around 9.5 billion USD and is expected to surpass 17.5 billion USD in 2025 (9). In China, the largest scallop market, the preference of consumers from first-tier cities (e.g., Shanghai and Guangzhou) for scallops is mainly affected by the origin of the scallops rather than nutritional value, and imported shellfish from developed countries, such as the USA and Canada, are highly preferred (10). This consumer behavior is mainly due to higher trust in the food standards of more developed countries than in their own country’s food standards (11).

Proteins and lipids are the two most valuable nutrients in seafood (12, 13). On the one hand, amino acids (AAs) are the basic units of protein, and some AAs (essential AAs, EAAs) cannot be synthesized by the human body in adequate amounts, making diet the main source of these EAAs to maintain optimal health (14). On the other hand, long-chain polyunsaturated fatty acids (LC-PUFA), especially eicosapentaenoic acid (EPA) and docosahexaenoic acid (DHA), play an important role in fetal development, cardiovascular protection, inflammation modulation, etc. (15). Thus, seafood offers a unique combination of both EAAs and LC-PUFA, making it an essential food group for human nutrition and disease prevention.

Currently, there is a lack of comprehensive comparison of the nutritional values of scallops from different species and geographical origins, although there are several general reviews on bivalve proteins (7, 16, 17) and lipids (18–20). The objective of this study is to evaluate the quality of amino acids and fatty acids in scallops based on secondary data from relevant published articles. The findings of this study not only provide consumers with comprehensive information on the protein and lipid quality of edible scallops on a global scale, but also provide important information to scientists and aquaculturists for choosing scallop varieties for selective breeding.

## 2 Materials and methods

### 2.1 Literature search

Relevant articles were retrieved from Google Scholar, PubMed, Scopus, and Web of Science using a combination of the following keywords: “scallops,” “proximate composition,” “proteins,” “lipids,” “amino acids,” “fatty acids,” “essential amino acids,” “amino acid score,” “EPA + DHA,” and “PUFA.” The reference lists of all articles were carefully screened to identify any additional studies relevant to this study. This process was repeated until no new articles could be identified. As a result, a total of 2,871 articles were initially retrieved, covering relevant literature published up to 30th May 2025.

### 2.2 Screening and exclusion process

All retrieved articles were manually screened to exclude those that did not provide complete proximate composition, amino acid profiles, or fatty acid profiles in tabular form or supplementary tables. After full-text assessment, 91 articles met all inclusion criteria and were retained for subsequent data acquisition.

### 2.3 Data acquisition and statistical analysis

From each of the 91 included articles, key information was recorded, including common name, scientific name, location, geographical climate, proximate composition, amino acid indices, and fatty acid indices of the sampled scallops. Given the high variability in moisture content among samples, all proximate composition data were converted into a dry weight basis for better comparison.

For protein quality assessment, total amino acid and total essential amino acid data were standardized to g/ 100 g protein. AA scores were calculated according to the FAO/WHO pattern (21). For lipid quality assessment, the EPA + DHA content, PUFA/SFA ratio, and n-3/n-6 ratio were calculated and expressed as percentages of total fatty acids (%). Given the huge variation in nutrient composition among distinct scallop samples, the present review mostly conducted only descriptive analysis. Microsoft Excel was used for data organization and calculation of ranges, medians, means, and standard deviations.

## 3 Results

### 3.1 Proximate compositions of scallops

The proximate composition of scallops is summarized in Table 1. Across the literature, scallop moisture content ranged from 76.50 to 83.00%, protein content from 59.02 to 86.70% DW, lipid content from 0.48 to 20.77% DW, carbohydrate content from 1.42 to 25.83% DW, and ash content from 4.49 to 8.30% DW. Crude fiber content has not been determined in any species of scallop.

**Table 1:** Proximate composition of commercially important marine scallops.

Common name	Scientific name	Moisture (WW)	Protein (DW)	Lipid (DW)	Carbohydrate (DW)	Ash (DW)	Location	Region	References
Asian moon scallop	*Amusium pleuronectes*	82.50	81.71	2.29	25.83	7.09	Kedung, Jepara	Tropical	(33)
Yesso scallop	*Patinopecten yessoensis*	-	64.59	16.08	-	-	Ailian Bay, China	Temperate	(26)
Zhikong scallop	*Chlamys farreri*	-	68.65	20.07	-	-	Ailian Bay, China	Temperate	(26)
Zhikong scallop	*Chlamys farreri*	81.79	70.82	19.38	-	-	Shandong, China	Temperate	(27)
Golden scallop	*Chlamys nobilis*	69.68	69.53	14.02	-	4.49	Nan’ao, China	Subtropical	(32)
Yellow scallop	*Chlamys nobilis*	69.61	69.56	14.08	-	4.54	Nan’ao, China	Subtropical	(32)
Brown scallop	*Chlamys nobilis*	70.62	68.18	13.24	-	4.66	Nan’ao, China	Subtropical	(32)
Zhikong scallop	*Chlamys farreri*	81.49	71.20	17.50	-	-	Shandong Peninsular	Temperate	(36)
Chilean scallop	*Argopecten purpuratus*	-	72.23	-	-	-	Sechura Bay, Peru	Tropical	(29)
Great Mediterranean Scallop	*Pecten jacobaeus*	79.42	61.08	9.86	-	6.71	Gulf of Antalya	Subtropical	(30)
Golden scallop	*Chlamys nobilis*	-	-	0.50	-	-	Nan’ao Island	Subtropical	(28)
Brown scallop	*Chlamys nobilis*	-	-	0.48	-	-	Nan’ao Island	Subtropical	(28)
Zhikong scallop	*Chlamys farreri*	-	-	5.09	-	-	Liaodong, China	Temperate	(35)
Yesso scallop	*Patinopecten yessoensis*	-	-	4.08	-	-	Liaodong, China	Temperate	(35)
Farmed rock scallop	*Crassadoma gigantea*	77.45	73.75	1.64	-	7.45	Vancouver, Canada	Temperate	(31)
Smooth scallop	*Flexopecten glaber*	83.00	65.65	11.35	14.29	8.76	Canakkale, Turkey	Subtropical	(62)
Pacific rock scallop	*Crassadoma gigantea*	78.68	75.66	4.32	9.01	8.30	Vancouver, Canada	Temperate	(24)
Atlantic bay scallop	*Patiopecten irradians*	79.13	77.67	5.94	8.54	7.20	Dalian, China	Temperate	(24)
Atlantic bay scallop	*Argopecten irradians*	79.15	67.29	5.76	14.72	7.63	Dalian, China	Temperate	(24)
Zhikong scallop	*Chlamys farreri*	81.81	83.01	5.88	2.47	7.31	Dalian, China	Temperate	(24)
Noble scallop	*Chlamys nobilis*	78.12	83.14	2.83	1.42	5.85	Zhanjiang, China	Tropical	(23)
King scallop	*Pecten maximus*	78.20	86.70	5.05	12.39	6.42	Norway	Temperate	(22)
King scallop	*Pecten maximus*	76.50	85.11	4.26	14.89	6.38	France	Subtropical	(22)
Atlantic bay scallop	*Argopecten irradians*	81.27	67.81	11.8	-	7.74	Qinhuangdao, China	Temperate	(25)
Noble scallop	*Chlamys nobilis*	82.04	59.02	20.77	-	6.68	Qinhuangdao, China	Temperate	(25)
Chilean scallop	*Argopecten purpuratus*	-	-	3.27	-	-	Calbuci, Chile	Temperate	(63)
Atlantic deep sea scallop	*Placopecten magellanicus*	-	-	2.01	-	-	Sandy Cove, Nova Scotia	Temperate	(38)

The highest documented scallop protein content (85.11 to 86.70% DW) was found in *Pecten maximus* from Norway and France (22), followed by *Chlamys nobilis* (83.14% DW) from Zhanjiang, China (23), and *Chlamys farreri* (83.01% DW) from Dalian, China (24). Conversely, the lowest protein content was recorded for *Chlamys nobilis* (59.02% DW) from Qinhuangdao, China (25).

The highest recorded scallop lipid content was documented in *Chlamys nobilis* (20.77% DW) from Qinhuangdao, China (25), followed by *Chlamys farreri* (20.07% DW) from Ailian Bay, China (26), and *Chlamys farreri* (19.38% DW) from Shandong, China (27). The lowest lipid contents were recorded in *Chlamys nobilis* (0.48 to 0.50% DW) from Nan’ao Island, China (28).

### 3.2 Protein quality of scallops

The total amino acid (TAA) content, essential amino acid (EAA) content, and EAA/TAA ratio of scallops are summarized in Table 2. The TAA of scallops ranged from 22.36 to 96.49 g/100 g protein, with the highest TAA content recorded in *Argopecten irradians* (96.49 g/100 g protein) from Qinhuangdao, China (25), followed by *Chlamys farreri* (83.62 g/100 g protein) from Dalian, China (24), and *Patinopecten yessoensis* (75.17 g/100 g protein) from Ailian Bay, China (26).

**Table 2:** Amino acids and essential amino acid content in commercially important marine scallops.

Common name	Species	Location	Region	Total AA (g/100 g protein)	EAA (g/100 g protein)	E/T (%)	Major 5 AA	Taurine (g/ 100 g dry weight)	References
Yesso scallop	*Patinopecten yessoensis*	Ailian Bay, China	Temperate	75.17	22.73	30.24	Arg>Asp> Glu>Lys>Gly	2.12	(26)
Zhikong scallop	*Chlamys farreri*	Ailian Bay, China	Temperate	73.37	23.02	31.38	Arg>Asp> Glu>Lys>Leu	2.33	(26)
Zhikong scallop	*Chlamys farreri*	Shandong, China	Temperate	63.16	19.52	30.91	Gly>Arg>Asp> Glu>Ala	-	(27)
Golden scallo	*Chlamys nobilis*	Nan’ao Island, China	Subtropical	42.1	11.82	28.08	Glu>Gly>ASP> Arg>Lys	-	(32)
Yellow scallop	*Chlamys nobilis*	Nan’ao Island, China	Subtropical	45.63	13.52	29.63	Glu>Gly>ASP> Lys>Arg	-	(32)
Brown scallop	*Chlamys nobilis*	Nan’ao Island, China	Subtropical	48.73	13.81	28.34	Glu>Gly>ASP> Lys>Arg	-	(32)
Zhikong scallop	*Chlamys farreri*	Shandong Peninsular, China	Temperate	53.83	21.17	39.33	-	-	(36)
Chilean scallop	*Argopecten purpuratus*	Sechura Bay, Peru	Tropical	69.18	25.99	37.57	Glu>Gly>Asp> Arg>Leu	-	(29)
Scallop	*Mimachlamys nobilis*	Zhejiang, China	Subtropical	38.06	17.83	46.85	Glu>Gly>Asp> Lys>Leu>	-	(64)
Great Mediterranean Scallop	*Pecten jacobaeus*	Gulf of Antalya, Turkey	Subtropical	22.36	12.14	54.29	Asp> Glu>Lys>Thr>Leu	-	(30)
Golden scallop	*Chlamys nobilis*	Nan’ao, China	Subtropical	57.21	19.82	34.64	Glu>Gly>Arg>Asp> Lys	2.83	(65)
Brown scallop	*Chlamys nobilis*	Nan’ao, China	Subtropical	68.25	23.99	35.15	Glu>Gly>Arg>Asp> Leu	4.21	(65)
Farmed Pacific rock scallop	*Crassadoma gigantea*	Vancouver, Canada	Temperate	66.32	25.62	38.63	Glu>Gly>Leu>Arg>Lys	-	(31)
Pacific rock scallop	*Crassadoma gigantea*	Vancouver, Canada	Temperate	68.25	24.25	35.54	Glu>Asp> Leu>Gly>Arg	-	(24)
Atlantic bay scallop	*Patiopecten irradians*	Dalian, China	Temperate	65.45	21.90	33.47	Glu>Gly>Asp> Arg>Leu	-	(24)
Atlantic bay scallop	*Argopecten irradians*	Dalian, China	Temperate	58.33	18.23	48.18	Glu>Gly>Arg>Asp> Leu	-	(24)
Zhikong scallop	*Chlamys farreri*	Dalian, China	Temperate	83.62	28.59	55.66	Glu>Asp> Gly>Arg>Leu	-	(24)
Noble scallop	*Chlamys nobilis*	Zhanjiang, China	Tropical	50.37	20.91	41.52	Glu>Asp> Lys>Leu>Val	-	(23)
Atlantic bay scallop	*Argopecten irradians*	Qinhuangdao, China	Temperate	96.49	29.41	30.48	Glu>Asp> Gly>Arg>Leu	-	(25)

The EAA content of scallops ranged from 11.82 to 29.41 g/100 g protein, with the highest EAA content recorded in *Argopecten irradians* (29.41 g/100 g protein) from Qinhuangdao, China (25), followed by *Chlamys farreri* (28.59 g/100 g protein) from Dalian, China (24), and *Argopecten purpuratus* (25.99 g/100 g protein) from Sechura Bay, Peru (29). The E/T ratio of scallops ranged from 28.08 to 55.66%, with the highest E/T ratio recorded in *Chlamys farreri* (55.66%) from Dalian, China (24), followed by Pecten jacobaeus (54.29%) from the Gulf of Antalya, Turkey (30) and *Argopecten irradians* (48.18%) from Dalian, China (24).

Taurine content of scallops ranged from 2.12 to 4.21 g/100 g DW, and this metabolite was not reported in most studies. The amino acid scores for scallop EAAs are summarized in Table 3. In general, one or more EAAs were limiting in the evaluated scallops. Farmed *Crassadoma gigantea* from Vancouver, Canada was the best source of EAAs, with scores for all EAAs exceeding 90% and most exceeding 100% (31). Wild *Crassadoma gigantea* from Vancouver, Canada (31) and *Chlamys farreri* from Dalian, China (24) were also good sources of EAAs, with all EAA scores above 80% and most above 100%. However, certain EAAs, especially Val, Cys, and Met, were critically limited in some scallops (26, 27, 32, 33).

**Table 3:** Amino acid scores of commercially important marine scallops.

Common name	Species	Location	Region	Thr (g/100 g protein)	Cys+Met (g/ 100 g dry weight)	Val (g/ 100 g dry weight)	Ile (g/ 100 g dry weight)	Leu (g/ 100 g dry weight)	Phe+Tyr (g/ 100 g dry weight)	Lys (g/ 100 g dry weight)	References
Asian moon scallop	*Amusium pleuronectes*	Kedung, Jepara	Tropical	96.37	281.14	24.68	96.37	113.98	78.96	160.73	(33)
Yesso scallop	*Patinopecten* *yessoensis*	Ailian Bay, China	Temperate	114.74	71.46	34.59	87.47	89.32	70.29	184.07	(26)
Zhikong scallop	*Chlamys farreri*	Ailian Bay, China	Temperate	134.94	70.48	36.80	92.00	97.16	73.83	174.95	(26)
Zhikong scallop	*Chlamys farreri*	Shandong, China	Temperate	116.77	55.19	80.75	103.31	82.10	103.70	136.16	(27)
Iceland scallop	*Chlamys islandica*	Dalnezelenetskaya Bay, Russia	Temperate	165.20	-	209.25	126.65	92.83	141.34	72.09	(66)
Golden scallop	*Chlamys nobilis*	Nan’ao Island, China	Subtropical	85.41	60.13	45.10	101.38	77.06	108.58	136.28	(32)
Yellow scallop	*Chlamys nobilis*	Nan’ao Island, China	Subtropical	85.14	85.51	35.22	125.70	79.08	118.67	133.35	(32)
Brown scallop	*Chlamys nobilis*	Nan’ao Island, China	Subtropical	87.82	82.72	34.97	112.95	84.25	117.93	138.22	(32)
Chilean scallop	*Argopecten purpuratus*	Sechura Bay, Peru	Tropical	3.19	2.89	2.28	2.62	4.52	4.22	3.61	(29)
Great Mediterranean Scallop	*Pecten jacobaeus*	Gulf of Antalya, Turkey	Subtropical	271.70	131.82	62.39	73.42	116.23	205.66	223.03	(30)
Golden scallop	*Chlamys nobilis*	Nan’ao Island, China	Subtropical	104.29	53.04	82.18	96.99	112.78	105.85	155.29	(65)
Brown scallop	*Chlamys nobilis*	Nan’ao Island, China	Subtropical	103.02	57.02	82.94	98.70	113.92	109.58	155.35	(65)
Farmed Pacific rock scallop	*Crassadoma gigantea*	Vancouver, Canada	Temperate	98.35	110.36	92.99	121.60	127.73	140.68	146.96	(31)
Pacific rock scallop	*Crassadoma gigantea*	Vancouver, Canada	Temperate	113.40	100.15	86.60	109.97	115.86	131.73	127.46	(24)
Atlantic bay scallop	*Patiopecten irradians*	Dalian, China	Temperate	106.15	98.31	79.06	109.81	110.86	112.25	121.12	(24)
Atlantic bay scallop	*Argopecten irradians*	Dalian, China	Temperate	98.20	88.85	75.29	98.20	101.71	109.11	116.05	(24)
Zhikong scallop	*Chlamys farreri*	Dalian, China	Temperate	108.48	93.92	81.53	103.55	109.89	121.63	127.91	(24)
Noble scallop	*Chlamys nobilis*	Zhanjiang, China	Tropical	130.38	74.04	120.81	125.85	109.40	149.36	170.45	(23)
Zhikong scallop	*Chlamys farreri*	Qingdao, China	Temperate	99.56	63.21	88.50	77.43	94.82	78.66	101.90	(67)
Atlantic bay scallop	*Argopecten irradians*	Qingdao, China	Temperate	93.75	107.14	94.44	79.86	89.29	69.44	101.01	(67)
Yesso scallop	*Patiopecten yessoensis*	Qingdao, China	Temperate	92.47	56.61	87.19	82.56	86.81	68.25	278.61	(67)
Noble scallop	*Chlamys nobilis*	Qingdao, China	Temperate	93.53	65.78	126.62	89.93	94.55	76.74	316.55	(67)
Atlantic (bay scallop)	*Argopecten irradians*	Qinhuangdao, China	Temperate	112.19	102.45	80.01	95.86	111.93	63.22	13.38	(25)

### 3.3 Lipid quality of scallops

The lipid quality indices of scallops are summarized in Table 4. The EPA + DHA content in scallops ranged from 6.93 to 52.00% of total fatty acids. The highest EPA + DHA content was recorded in *Nodipecten subnodosus* (52.00%) from Guerrero Negro, Mexico (34), followed by *Pecten maximus* from Kvitsoy, Norway (51.30%) and Somna, Turkey (50.50%) (34).

**Table 4:** Lipid indices in commercially important marine scallops.

Common name	Species	EPA+DHA (%)	n-3/n-6	PUFA/SFA	Location	Region	References
Yesso scallop	*Patinopecten* *yessoensis*	47.83	5.00	1.86	Ailian Bay, China	Temperate	(26)
Zhikong scallop	*Chlamys farreri*	45.10	8.50	1.60	Ailian Bay, China	Temperate	(26)
Golden noble scallop	*Chlamys nobilis*	26.33	6.08	0.61	Nan’ao Island, China	Subtropical	(32)
Yellow noble scallop	*Chlamys nobilis*	26.38	5.54	0.63	Nan’ao Island, China	Subtropical	(32)
Brown noble scallop	*Chlamys nobilis*	22.55	4.62	0.52	Nan’ao Island, China	Subtropical	(32)
Zhikong scallop	*Chlamys farreri*	47.66	8.00	4.35	Shandong Peninsular, China	Temperate	(36)
Golden noble scallop	*Chlamys nobilis*	36.70	5.78	1.08	Nan’ao Island, China	Subtropical	(28)
Brown noble scallop	*Chlamys nobilis*	34.40	5.63	0.93	Nan’ao Island, China	Subtropical	(28)
Variegated scallop	*Mimachlamys* var*ia*	27.40	8.05	1.15	Gulf of Taranto, Italy	Subtropical	(68)
Smooth scallop	*Flexopecten glaber*	18.27	2.70	1.01	Ionian Sea, Albania	Temperate	(69)
Smooth scallop	*Flexopecten glaber*	9.79	3.10	1.19	Ionian Sea	Temperate	(69)
Zhikong scallop	*Chlamys farreri*	29.32	19.88	2.07	Liaodong, China	Temperate	(35)
Yesso scallop	*Patinopecten yessoensis*	30.45	20.18	1.94	Liaodong, China	Temperate	(35)
Variegated scallop	*Mimachlamys* var*ia*	22.60	6.65	0.93	Gulf of Taranto, Italy	Subtropical	(70)
Smooth scallop	*Flexopecten glaber*	12.80	2.66	0.51	Gulf of Taranto, Italy	Subtropical	(70)
Variegated scallop	*Chlamys varia*	-	1.41	0.05	Barbariga Cape, Croatia	Temperate	(54)
Variegated scallop	*Chlamys varia*	-	-	0.01	Barbariga Cape	Temperate	(54)
Variegated scallop	*Chlamys varia*	-	-	0.01	Barbariga Cape	Temperate	(54)
Variegated scallop	*Chlamys varia*	-	-	0.03	Barbariga Cape	Temperate	(54)
Smooth scallop	*Flexopecten glaber*	8.00	2.70	0.24	Gulf of Taranto	Temperate	(71)
Smooth scallop	*Flexopecten glaber*	17.20	4.73	0.71	Gulf of Taranto	Temperate	(71)
Smooth scallop	*Flexopecten glaber*	21.40	5.37	0.90	Gulf of Taranto, Italy	Temperate	(71)
Smooth scallop	*Flexopecten glaber*	19.10	4.49	0.68	Gulf of Taranto	Temperate	(71)
Smooth scallop	*Flexopecten glaber*	15.68	3.47	0.56	Gulf of Taranto, Italy	Temperate	(72)
Variegated scallop	*Mimachlamys varia*	24.87	6.57	1.06	Gulf of Taranto, Italy	Temperate	(72)
Farmed Pacific rock scallop	*Crassadoma gigantea*	45.20	11.42	1.51	Vancouver, Canada	Temperate	(31)
Smooth scallop	*Flexopecten glaber*	26.61	6.75	0.70	Ionian Sea, Albania	Temperate	(68)
Variegated scallop	*Mimachlamys varia*	35.23	6.77	1.47	Ionian Sea, Albania	Temperate	(68)
Mediterranean scallop	*Pecten jacobaeus*	27.64	7.40	0.87	Ionian Sea, Albania	Temperate	(68)
Pacific rock scallop	*Crassadoma gigantea*	41.84	7.11	1.56	Vancouver, Canada	Temperate	(24)
Atlantic bay scallop	*Patiopecten irradians*	45.77	7.73	1.62	Dalian, China	Temperate	(24)
Atlantic bay scallop	*Argopecten irradians*	39.90	4.47	1.53	Dalian, China	Temperate	(24)
Zhikong scallop	*Chlamys farreri*	41.26	11.84	1.53	Dalian, China	Temperate	(24)
Yesso scallop	*Patinopecten yessoensis*	33.70	5.25	2.17	Peter the Great Bay, Russia	Temperate	(73)
King scallop	*Pecten maximus*	39.30	9.47	1.50	Bay of Brest, France	Temperate	(74)
King scallop	*Pecten maximus*	46.20	10.90	1.89	Frøya and Hitra, Norway	Temperate	(22)
King scallop	*Pecten maximus*	43.90	10.70	1.68	Brittany and Normandy coast, France	Temperate	(22)
King scallop	*Pecten maximus*	40.90	15.20	1.63	Frozen	-	(22)
Deep sea scallop	*Placopecten magellanicus*	41.60	18.20	1.69	Frozen	-	(22)
Smooth scallop	*Flexopecten glaber*	5.80	2.54	0.31	Bizerte lagoon, Tunisia	Subtropical	(55)
Atlantic bay scallop	*Argopecten irradians*	30.15	7.24	1.06	Qinhuangdao, China	Temperate	(25)
Mediterranean scallop	*Pecten jacobaeus*	22.50	5.27	0.89	Kelibia, Tunisia	Subtropical	(56)
Variegated Scallop	*Chlamys varia*	30.50	9.04	1.10	Bizerte lagoon, Tunisia	Subtropical	(56)
Smooth scallop	*Flexopecten glaber*	6.93	7.51	0.31	Bizerte lagoon, Tunisia	Subtropical	(56)
King scallop	*Pecten maximus*	47.40	15.00	1.87	Kvitsoy, Norway	Temperate	(35)
King scallop	*Pecten maximus*	51.30	11.65	1.95	Kvitsoy, Norway	Temperate	(34)
King scallop	*Pecten maximus*	47.50	12.32	1.75	Askvoll, Norway	Temperate	(34)
King scallop	*Pecten maximus*	48.60	12.93	1.82	Askvoll, Norway	Temperate	(34)
King scallop	*Pecten maximus*	47.80	15.44	1.75	Sandoy, Denmark	Temperate	(34)
King scallop	*Pecten maximus*	48.40	13.36	1.80	Sandoy, Denmark	Temperate	(34)
King scallop	*Pecten maximus*	47.90	14.15	1.77	Froya, Norway	Temperate	(34)
King scallop	*Pecten maximus*	47.50	14.44	1.80	Froya, Norway	Temperate	(34)
King scallop	*Pecten maximus*	47.30	14.89	1.73	Somna, Turkey	Temperate	(34)
King scallop	*Pecten maximus*	50.50	12.42	1.90	Somna, Turkey	Temperate	(34)
Zhikong scallop	*Chlamys farreri*	17.00	5.73	1.55	Jiaozhou Bay, China	Subtropical	(75)
Giant lion’s paw	*Nodipecten subnodosus*	37.50	8.38	2.25	Guerrero Negro	Subtropical	(37)
Giant lion’s paw	*Nodipecten subnodosus*	52.00	10.85	3.40	Guerrero Negro, Mexico	Subtropical	(37)
Giant lion’s paw	*Nodipecten subnodosus*	5.00	10.00	3.17	Guerrero Negro	Subtropical	(37)
Chilean scallop	*Argopecten purpuratus*	42.00	12.10	2.33	Calbucco, Chile	Temperate	(76)
Chilean scallop	*Argopecten purpuratus*	42.00	12.10	1.96	Calbuci, Chile	Temperate	(63)
Atlantic deep sea scallop	*Placopecten magellanicus*	32.00	9.44	2.59	Sandy Cove, Nova Scotia	Temperate	(38)
Atlantic deep sea scallop	*Placopecten magellanicus*	45.92	12.98	2.56	Southeastern Newfoundland	Temperate	(77)
-	*Chlamys tehuelcha*	15.30	7.78	1.35	San Jose Gulf, Argentina	Temperate	(57)

The n-3/n-6 ratio in scallops ranged from 1.41 to 20.18. The highest n-3/n-6 ratio was recorded in *Patinopecten yessoensis* (20.18) from Liaodong, China (35), followed by *Chlamys farreri* (19.88) from Liaodong, China (35), and *Placopecten magellanicus* (18.20%) (22).

The PUFA/SFA ratio in scallops ranged from 0.05 to 4.35. The highest value was recorded in *Chlamys farreri* (4.35) from Shandong, China (36), followed by *Nodipecten subnodosus* (3.40) from Guerrero Negro, Mexico (37), and *Placopecten magellanicus* (2.59) from Sandy Cove, Nova Scotia (38).

## 4 Discussion

Proteins and lipids are among the most valuable macronutrients in foods, providing substantial health advantages and versatile applications in nutrition (39). According to Stansby and Olcott (40), most scallops are classified as low oil-high protein foods (lipid < 5%; protein = 15–20% WW), with two exceptions classified as low oil-very high protein (lipid < 5%; protein >20% WW): *Chlamys nobilis* from Nan’ao, China (32) and *Pecten maximus* from France (22). However, the high protein content recorded for *Chlamys nobilis* from Nan’ao, China, was likely due to an unusually low moisture content (69–61 to 70.62%) (32), suggesting that using dry weight to evaluate seafood nutritional value is more reliable. Interestingly, the lipid content of scallops is significantly influenced by their geographical region. For example, the lipid content in *Pecten maximus* from temperate regions was higher than in those from subtropical regions (22). A similar pattern is observed in *Chlamys* spp., where the lipid content of *Chlamys farreri* from temperate waters (24, 26, 27, 35) was significantly higher than that of *Chlamys nobilis* from subtropical waters (23, 28, 32). These observations are attributed to the role of higher lipid reserves in providing energy for metabolic maintenance, maintaining membrane fluidity, and resisting oxidative stress (41, 42).

The TAA and EAA contents of scallops (TAA = 60.90 ± 16.89%, ranged from 22.36 to 96.49%, median = 63.16; EAA = 21.00 ± 5.21%, ranged from 11.82 to 29.41%, median = 21.17%; E/T = 37.27 ± 8.23%, ranged from 28.08 to 55.66%, median = 35.15%) are superior to those of shrimp (TAA = 20.88 to 21.94%; EAA = 10.71 to 11.88%; E/T = 45.00 to 49.00%) (43) but are comparable to or lower than those of terrestrial livestock (beef, pork, lamb etc.) (TAA = 50.50 to 93.60; EAA = 37.30 to 42.50%; E/T = 44.02 to 73.86%) (44), fish (TAA = 41.57 to 45.76; EAA = 17.79 to 19.13%; E/T = 41.72 to 42.72%) (45), and marine crabs and gastropods (TAA = 60.45 to 87.70; EAA = 26.64 to 40.40%; E/T = 43.18 to 46.07%) (46, 47), indicating that scallops are a good source of animal protein. The taurine content in scallops ranged from 2.12 to 4.21 g/100 g DW. These values are higher than those reported for many commercially important fish (0.03 to 0.18 g/100 g WW), including redfish, whiting, salmon, ray, and tuna (48). Although taurine offers many health benefits (49), its content in scallops is not commonly reported. Therefore, future studies on scallop protein quality should also include taurine analysis.

Amino acid scoring revealed that very few scallops have complete EAAs that align with human nutritional requirements (24, 31). One or more EAAs are limited in most scallops, with Val frequently being the primary limitation, as reported in *Amusium pleuronecte*s from Kedung, Jepara (33), *Patinopecten yessoensis* from Ailian Bay, China (26), *Chlamys farreri* from Ailian Bay, China (26), *C. nobilis* from Nan’ao Island (32), and *Pecten jacobaeus* from Gulf of Antalya, Turkey (30). Likewise, Val is a major limited EAA in crabs (46) and fish (45), with additional limitations in Cys, Ile, Leu, and etc. (45). These observations suggest that scallops provide a relatively complete and balanced EAA profile compared to many other seafood sources.

Scallop lipid quality (EPA + DHA = 33.38 ± 13.53%; n-3: n-6 = 8.84 ± 4.42; PUFA/SFA = 1.41 ± 0.82) was relatively higher than that of many marine and freshwater finfish (EPA + DHA = 15.00 to 17.20; n-3: n-6 = 2.60 to 9.03; PUFA/SFA = 0.96 to 1.29) (50, 51) and invertebrates (e.g., shrimps and sea cucumbers) (6.29 to 18.24%, n-3, n-6 = 1.25 to 8.33, PUFA/SFA = 0.55 to 1.29) (43, 52, 53). These indices are comparable to those of other high-quality seafood, including oysters (EPA + DHA = 26.22 ± 10.76%; n-3: n-6 = 5.74 ± 2.87; PUFA/SFA = 1.36 ± 0.79), krill (EPA + DHA = 24.00%; n-3: n-6 = 13.00; PUFA/SFA = 1.35) (51), and high lipid quality fish (sardines, menhaden, and anchoveta) (EPA + DHA = 27.00 ± 4.58%; n-3: n-6 = 10.56 ± 0.96; PUFA/SFA = 1.03 ± 0.18). Collectively, these observations indicate that scallop possess superior lipid quality. However, it is worth noting that some scallops have a PUFA/SFA ratio below 0.45, including *Chlamys* var*ia* from Barbariga Cape, Croatia (54) and *Flexopecten glaber* from Bizerte lagoon, Tunisia (55, 56). This suggests that consumers should consider these variations when making dietary choices.

It is worth noting that the lipid quality of scallops is highly dependent on geographical region. Within the same *Chlamys* genus, scallops inhabiting temperate regions (e.g., *Chlamys farreri* and *Chlamys tehuelcha*) generally exhibit higher levels of EPA + DHA, n-3: n-6, and PUFA/SFA than those from subtropical regions (e.g., *Chlamys nobilis, Chlamys varia*) (24, 26, 28, 32, 35, 36, 57). This observation could be attributed to a combination of environmental, dietary, and physiological factors (58, 59). On the one hand, the phytoplankton food source available to scallops in temperate regions contain higher concentrations of n-3 LC-PUFA due to the activation of desaturase enzymes involved in EPA and DHA synthesis (60). On the other hand, bivalves increase membrane fluidity in low-temperature environments by retaining higher levels of n-3 LC-PUFA, especially EPA and DHA (41). However, we cannot rule out the possibility that these differences were influenced by species-specific factors, as scallops, unlike other bivalves, have very clear geographical boundary due to their narrow temperature tolerance (61).

Caution is warranted, as this study has several potential limitations. First, environmental factors (e.g., geographical regions, food availability, climate) and biological factors (e.g., species, age, size, reproductive stage) that significantly influence the nutritional composition of scallops could not be fully controlled or accounted for, as most of this information was not provided in the published literature. Second, reliance on secondary data from published articles may introduce variability, as sampling procedures, analytical methods, and reporting standards were not completely identical across studies, ultimately affecting the consistency and comparability of the nutrient profiles. Third, relevant data published in languages other than English or not accessible through the Google Scholar, PubMed, Scopus, and Web of Science databases were not fully included in the present study, potentially leading to geographical biases. Fourth, most published studies did not report the reproductive status of analyzed scallops; some used whole soft tissue, while others used only the adductor muscle. This inconsistency hinders the comparison of nutrients, particularly lipid profiles.

## 5 Conclusion

In conclusion, the reported protein and lipid content of scallops varied considerably among studies. Scallops are generally a good source of proteins and lipids, with satisfactory protein quality and excellent lipid quality. Although some scallops have a good AA score for all EAAs, indicating they can provide consumers with all required EAAs, valine is the main limiting EAAs in many scallops. Regarding fatty acid profiles, scallops are rich in n-3 LC-PUFA, with EPA and DHA collectively accounting for over 20% of total fatty acids in most species, in some scallops, this figure reaches up to 50%. The lipid profile of scallops, reflected in their n-3/n-6 and PUFA/SFA ratios, generally exceeds recommended standards, with only rare exceptions showing unusually low PUFA/SFA levels. The findings of this study provide a comprehensive overview of the protein and lipid quality of edible scallops. Such information can serve as a guide for expanding and developing scallop aquaculture, as well as for conducting selective breeding programs for scallops. Future research should aim to standardize reporting metrics and expand data inclusion to cover studies published in different languages and available across various platforms, thereby refining our understanding of scallops as a sustainable source of high-quality nutrition within a growing global food system.
